# Assessment of MLC tracking performance during hypofractionated prostate radiotherapy using real-time dose reconstruction

**DOI:** 10.1088/0031-9155/61/4/1546

**Published:** 2016-01-27

**Authors:** M F Fast, C P Kamerling, P Ziegenhein, M J Menten, J L Bedford, S Nill, U Oelfke

**Affiliations:** Joint Department of Physics, The Institute of Cancer Research and The Royal Marsden NHS Foundation Trust, London SM2 5NG, UK; martin.fast@icr.ac.uk; corijn.kamerling@icr.ac.uk

**Keywords:** MLC tracking, dose reconstruction, dose accumulation, prostate cancer, hypofractionation

## Abstract

By adapting to the actual patient anatomy during treatment, tracked multi-leaf collimator (MLC) treatment deliveries offer an opportunity for margin reduction and healthy tissue sparing. This is assumed to be especially relevant for hypofractionated protocols in which intrafractional motion does not easily average out. In order to confidently deliver tracked treatments with potentially reduced margins, it is necessary to monitor not only the patient anatomy but also the actually delivered dose during irradiation. In this study, we present a novel real-time online dose reconstruction tool which calculates actually delivered dose based on pre-calculated dose influence data in less than 10 ms at a rate of 25 Hz. Using this tool we investigate the impact of clinical target volume (CTV) to planning target volume (PTV) margins on CTV coverage and organ-at-risk dose. On our research linear accelerator, a set of four different CTV-to-PTV margins were tested for three patient cases subject to four different motion conditions. Based on this data, we can conclude that tracking eliminates dose cold spots which can occur in the CTV during conventional deliveries even for the smallest CTV-to-PTV margin of 1 mm. Changes of organ-at-risk dose do occur frequently during MLC tracking and are not negligible in some cases. Intrafractional dose reconstruction is expected to become an important element in any attempt of re-planning the treatment plan during the delivery based on the observed anatomy of the day.

## 1. Introduction

Dynamic multi-leaf collimator (MLC) tracking is an emerging form of adaptive radiotherapy suitable for tumours which are affected by intrafractional motion. On conventional C-arm linacs, the use of MLC tracking has been reported for a small number of prostate patients (Keall *et al* 2014, Colvill *et al* 2015) giving some early indications about clinical usefulness. The benefit of applying MLC tracking to more mobile tumour sites such as lung is hypothesised, but as of today not demonstrated in clinical studies. Additionally, a much larger body of literature ranging back almost 15 years is available (Keall *et al* 2001, Tacke *et al* 2010, Fast *et al* 2014), mostly dealing with the technicalities of how to implement MLC tracking for the different linac vendors. MLC tracking, in its currently prevalent form, is focussed on dynamically reshaping the treatment field in the beam’s-eye-view according to the actual recorded target motion. This is sometimes referred to as *translational* or *centroid* tracking. Popular target detection devices are linac-mounted x-ray imagers (Poulsen *et al* 2010, Fast *et al* 2012) and the electromagnetic transponder-based localisation system (Keall *et al* 2011, Krauss *et al* 2011), both relying on implanted markers. Newly emerging, non-invasive and yet largely untested (in the context of radiotherapy) target detection techniques are ultrasound imaging (Schlosser *et al* 2010) and magnetic resonance (MR) imaging (Crijns *et al* 2012, Yun *et al* 2013). Especially the latter technique holds a lot of promise when available on a MR-guided delivery machine. Due to their superior soft-tissue contrast, MR images are expected to visualise not only target translations, but also target rotations and deformations as well as organ-at-risk (OAR) motion.

In this study, the focus will be on the dosimetric implications of dynamic MLC tracking during hypofractionated prostate irradiation. Currently, the calculation of actually delivered dose can only be conducted after the treatment fraction due to its computational cost, missing real-time data interfaces and compatibility issues with commercial treatment planning systems (TPS). Typical and well-publicised offline reconstruction techniques use a probability density function of the target position to either convolve the 3D dose or the 2D fluence (Lujan *et al* 1999, Waghorn *et al* 2010). As discussed by Poulsen *et al* (2012), these methods fail to correctly capture the machine/target motion interplay caused by adding dynamic MLC tracking. Alternatively, Poulsen *et al* (2012) propose the now frequently used rigid shift method: intrafractional target positions are grouped into discrete motion intervals, sub-beams are defined as part of the treatment corresponding to each motion interval, the target shift is modelled by rigidly shifting the sub-beam isocenter, and finally dose is calculated in a commercial TPS using a motion-encoded treatment plan. While this method correctly accounts for the machine/target motion interplay, the assumption that the anatomy surrounding the target is rigidly shifting in parallel with the target (thus invalidating the derived dose values for adjacent tissue that does not meet this condition) and the discretisation of motion could be seen as drawbacks. More recently, Ravkilde *et al* (2014) have presented an offline dose reconstruction algorithm specifically geared towards MLC tracking using a simplified pencil beam convolution algorithm.

To overcome the limitations of offline dose reconstruction, we are presenting a software solution which allows for real-time online dose reconstruction of dynamic radiotherapy deliveries. Online dose reconstruction is an essential link in the chain of online adaptive radiotherapy and the methodology developed for this study is expected to inform re-planning decisions impacting on the dose delivery at the time of treatment. In future, this might enable automated interventions with the possibility of updating treatment prescriptions before each beam in the case of step-and-shoot deliveries or even before each segment. Additionally, online dose reconstruction could potentially expedite the clinical adoption of MLC tracking by increasing confidence in the actually delivered dose. One of the main perceived advantages of MLC tracking is the ability to spare healthy tissue not only by continuously realigning the beam with the target, but also by shrinking the proportion of the treatment field margin directly related to interfractional and intrafractional motion. Using our new software, we investigated the impact of reduced margins on target coverage and OAR sparing.

## 2. Materials and methods

Dynamic MLC tracking was previously demonstrated at our institution for the Elekta Agility MLC (Stockholm, Sweden) hosted on a Synergy research linac (Fast *et al* 2014). While the previous study focussed on the technical aspects of realising MLC tracking such as system latencies and residual geometric errors, this study has seen our in-house developed control software for delivery and tracking, *DynaTrack*, expanded for full step-and-shoot IMRT deliveries. For the purpose of this study, a total of three delivery modes were implemented on our research linac.

• *Static*—no anatomical motion and no MLC tracking. The reconstructed dose is expected to be identical to the planned dose.
• *Conventional*—intrafractional anatomical motion, but no MLC tracking. This corresponds to the current standard of care. The reconstructed dose is expected to reveal changes in the target dose when compared to the *static* delivery. The effects of interfractional motion were also investigated for a small number of sample cases.
• *Tracked*—intrafractional target motion is compensated by MLC tracking. The reconstructed dose is expected to reveal that target dose is comparable and that OAR dose changes when comparing to the *static* delivery.

### 2.1. Online dose reconstruction software

**Figure 1.**
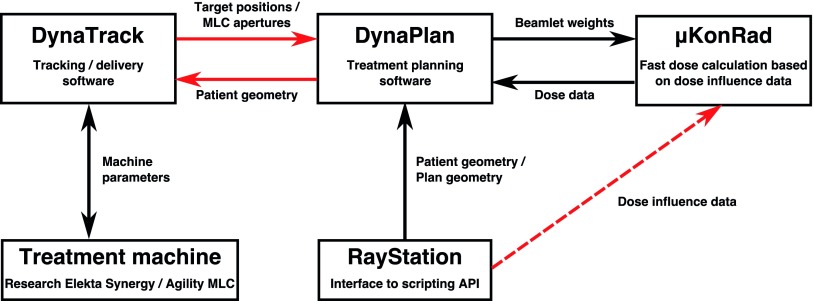
Schematic view of the online dose reconstruction platform. Connections for online use are shown as solid lines, offline connections are dashed. Interfaces explicitly implemented for this study are shown in red.

Our dose reconstruction software is schematically shown in figure 1. *DynaTrack* is interfaced to our research linac and controls treatment delivery and MLC tracking via a proprietary real-time tracking interface provided by Elekta. Actual MLC apertures and machine states are reported to *DynaTrack* every 40 ms and 20 ms respectively. Each MLC aperture is associated with the corresponding machine state (i.e. monitor units, dose rate, gantry angle and beam-on state) before being processed. Simulated 3D target positions based on measured prostate trajectories (see section 2.3) are updated at 25 Hz. An additional artificial latency of $100\pm 15$ ms is applied to the target positions to emulate the localisation latency of the Calypso system (Krauss *et al* 2011).

While *DynaTrack* is solely responsible for acquiring the target motion and controlling the treatment delivery, it is also connected to our in-house developed TPS, *DynaPlan*, via the TCP/IP networking protocol to facilitate dose reconstruction. For this study, *DynaPlan* was installed on a Windows 7 workstation computer equipped with two Intel Xeon E5-2697 v3 2.6 GHz CPUs in dual configuration. TCP/IP was chosen to allow *DynaTrack* and *DynaPlan* to run on different computers, and to ensure that each status package is actually sent and received. *DynaTrack* sends MLC apertures and target position updates to *DynaPlan* as soon as they become available using two independent TCP/IP connections. Separate connections were required as the data stem from two independent devices (linac and motion simulator) and are thus asynchronous by nature. To avoid a bunching of data, the TCP/IP receiver is organised in a separate thread independent of the computing-heavy dose reconstruction calculations. A third TCP/IP connection is used to send the content of the RT structure file to *DynaTrack* which is important for setting up the delivery correctly.

To facilitate dose accumulation, *DynaPlan* relies on the ultrafast *μKonRad* dose calculation engine (Ziegenhein *et al* 2008, 2013). *μKonRad* uses a set of pre-calculated dose influence matrices (${{D}_{\text{ij}}}$) generated in RayStation 4.6 (research version, RaySearch Laboratories, Stockholm, Sweden) using its singular value decomposed pencil beam algorithm to calculate the actually delivered dose. Beamlets were calculated independently for each beam for the CTV plus a 2.5 cm isotropic expansion to cover the range of anticipated target motion.

The main dose reconstruction thread continuously queries the receiver thread for new and unprocessed MLC apertures and performs the following tasks as soon as a new aperture becomes available:

(i) find the target position closest in time based on the data time stamps,
(ii) identify the corresponding set of beamlets based on the gantry angle,
(iii) assign weights to each beamlet according to its geometric overlap with the MLC,
(iv) start off a dose calculation for each beamlet,
(v) multiply each dose with the incremental MUs since the last reported aperture,
(vi) map the accumulated dose onto the planning CT (see section 2.3).

During step (vi), special consideration was given to the target volume-of-interest, which was mapped according to the target position associated with each aperture. In step (iii), special consideration was given to beamlets partially covered by the Y collimators and/or the leaves. For these beamlets, the weighting factor was linearly interpolated between the two states of closed (zero) and open (one). To avoid issues arising from the scheduler of the operating system and to balance the CPU load, steps (i) to (v) were also performed for apertures with incremental MU equal to zero i.e. beam-off. Although most measurements were performed as online dose reconstruction, it is important to note that certain additional batch calculations (i.e. rotations and interfractional motion, see section 2.3) were performed offline based on previously acquired online data. In these cases the large parameter space made online measurements impractical. For the offline mode, the dose reconstruction works in a similar way to the online mode outlined above by looping over the list of previously stored MLC apertures and target positions instead of requiring an active connection with *DynaTrack*.

A $5\times 5$ mm^2^ beamlet resolution was chosen for most measurements. The dose was accumulated onto a voxel grid derived from the planning CT by binning voxels by a factor of two in both transversal directions. The computed dose grid resolution was $2.3\times 2.3\times 1.5$ mm^3^ (patient 1), $2.1\times 2.1\times 1.5$ mm^3^ (patient 2) and $1.9\times 1.9\times 1.3$ mm^3^ (patient 3). The dose reconstruction was limited to slices 29–121 to reduce computation times. We have also tested a finer sampling (2.5 mm and 1.25 mm) in the direction parallel to the MLC leaves in offline reconstruction mode for a small subset of cases.

### 2.2. Patient cohort and planning rationale

To facilitate this investigation, we selected three typical prostate patients. The relevant patient characteristics are summarised in table 1. Note our use of V95% (total volume receiving 95% of the prescribed dose) overlap with the OAR as a proxy for the target-to-OAR distance. While data for patient 1 was used in a previous trial in Sydney (Ng *et al* 2012), data for patients 2 and 3 were newly acquired at our institute. All plans were specifically generated for this study according to the clinical dose constraints and fractionation scheme (5 Fx) outlined in the RTOG 0938 guidelines. The number of beams was 7 (patient 1 and 3) and 9 (patient 2). The standard 6 MV flattened beam was used for all deliveries. A total of four step-and-shoot IMRT plans were generated for each patient using (i) a RTOG-compliant CTV-to-PTV margin of 5 mm reduced to 3 mm posteriorly or (ii–iv) isotropic margins of 1 mm, 3 mm and 5 mm respectively. Plans were generated in Pinnacle (patient 1 and 3) and RayStation (patient 2).

**Table 1.** Patient cohort used in this study.

	CTV	PTV	Rectum V95%	Bladder V95%	NTID (litre × Gy)
	(cm^3^)				
*Patient #1*	55.4				
CTV+1 mm		70.4	0.5	12.7	37
CTV+3 mm		89.6	1.7	19.6	41.5
CTV+5 mm		111.5	2	25.1	46.5
CTV+5/3 mm		104.3	1.5	25.2	46
*Patient #2*	41.5				
CTV+1 mm		50.2	2.9	7.9	42
CTV+3 mm		69.1	3.1	12	47
CTV+5 mm		89.2	3.9	17.7	51
CTV+5/3 mm		84.5	2.9	16.9	50.5
*Patient #3*	51.6				
CTV+1 mm		66	1.8	7.6	38
CTV+3 mm		84.4	4	13.9	45
CTV+5 mm		110.4	6.1	17.6	50
CTV+5/3 mm		106.2	4.3	16.6	47

*Note*. Non-tumour integral dose (NTID) is introduced in section 2.4.

### 2.3. Motion conditions

To simulate intrafractional motion, we have identified four previously recorded prostate trajectories which represent a range of possible motion conditions including a baseline drift posteriorly and inferiorly (*continuous drift*), a baseline drift posteriorly and inferiorly with sudden transient motion mostly anteriorly (*erratic*), a slow baseline drift anteriorly and superiorly with sudden transient motion anteriorly and superiorly (*high frequency*), and a trajectory with little motion (*stable*). All of the trajectories were recorded for a previous study using the Calypso electromagnetic localisation system (Langen *et al* 2008). For a pictorial representation of the trajectories, see figures 1(a)–(c) (*continuous drift*, *high frequency* and *erratic*) and figure 3 (*stable*) in Langen *et al* (2008).

The motion trajectories were then used during the *conventional* and *tracked* deliveries to inform a simple motion model. The motion model assumes that the entire PTV is translated according to the trajectory, while the surrounding OARs remain static for want of a better OAR motion description. During dose accumulation, target dose was thus effectively mapped back into the target structure as defined on the planning CT. From a dose calculation point of view, the prostate motion model is justified based on the fact that the tissue surrounding the prostate is very homogenous and mostly of water-equivalent electron density, with the exception of pelvic bone which is sometimes quite near. To further underline the validity of our motion model, we have also implemented a second method similar to the rigid shift model introduced by Poulsen *et al* (2012). Here, instead of moving the target volume-of-interest (VOI) within the planning CT and the MLC aperture at the same time (‘MLC+VOI’), we keep the planning CT static and apply the residual shift of MLC motion minus target motion in beam’s-eye-view to the MLC (‘MLC-only’). The two methods were then compared for a small number of tracked deliveries.

Interfractional motion was only considered for a subset of the *conventional* deliveries by applying a constant offset vector to the intrafractional motion. This was done to estimate the impact of residual beam-to-target misalignment even after performing online image guidance (McNair *et al* 2008). Offset vectors from  −3 mm to  +3 mm in 1 mm steps in all three directions (and all combinations thereof resulting in 343 different dose reconstructions) were considered. For the tracked deliveries, the interfractional offset is implicitly corrected for and was thus not considered in this study.

Finally, prostate rotations were also applied on top of the translations during the reconstruction to investigate the potential impact on target coverage in *tracked* deliveries. Wu *et al* (2012) demonstrated how MLC tracking can accurately account for rotated targets but this was beyond the scope of this study. Instead, MLC tracking followed the translational motion of the target only. For all patients, pitch rotations about the left-right (LR) axis of the centre of volume, ranging from $-{{20}^{{}^\circ}}$ to $+{{20}^{{}^\circ}}$ in 1° steps, were investigated, since this type of rotation was found to be predominant in previous studies (Hoogeman *et al* 2005, Deutschmann *et al* 2012, Zhu *et al* 2013). Different combinations of pitch rotations with roll rotations about the superior-inferior (SI) axis, sampling the same angular range in 4° steps (121 different dose reconstructions), were additionally calculated, following the Euler convention of first pitch, then roll. Yaw rotations about the anterior-posterior (AP) axis were not investigated in this study. To exclude any interplay effects, the rotation was applied to the entire treatment fraction. Note that we also allowed for negative rotations in our study, as the planning CT might be a snapshot depicting a forward-rotated prostate.

### 2.4. Delivery analysis

The different delivery techniques, patient, plan and motion scenarios were compared by calculating dose volume histograms (DVH) for each VOI from the original treatment plans after completing the dose reconstruction. For these DVH calculations, all VOIs were treated as non-exclusive volumes meaning that some voxels were shared between volumes in the case of overlapping structures. From the DVHs, special points of interest such as CTV D98, PTV D95, rectum D2 and bladder D2 were then extracted. To compare the *static* treatment plans, the V95% for OARs and the non-tumour integral dose were computed. Following the definition by D’Souza and Rosen (2003) and assuming constant mass density ${{\rho}_{j}}$ across an organ *j*, the integral dose *I*_*j*_ to that organ is given by:

1 \begin{eqnarray*}{{I}_{j}}={{\rho}_{j}}\frac{{{V}_{j}}}{{{N}_{j}}}\underset{i}{\overset{{{N}_{j}}}{\mathop \sum}}\,{{D}_{i}},\end{eqnarray*}

where *V*_*j*_ is the volume and *N*_*j*_ is the number of voxels *i* with dose *D*_*i*_. The non-tumour ‘organ’ was derived by subtracting the CTV from the body VOI.

## 3. Results

Some of the dose characteristics of the different treatment plans are characterised in table 1. As expected, the overlap of V95% and OARs increases with margin. Although the overlap is also influenced by the conformity of the plan and generally by the clinical constraints on the optimiser, it indicates that the rectum-to-target distance is shortest for patient 1 and that the bladder-to-target distance is shortest (jointly) for patients 2 and 3. The non-tumour integral dose (1) is almost identical for patients 1 and 3 and slightly increased for patient 2, for which 9 beams were used instead of 7. Again as expected, an increase of non-tumour integral dose with margin is observed.

### 3.1. Dosimetric impact of intrafractional motion

**Figure 2.**
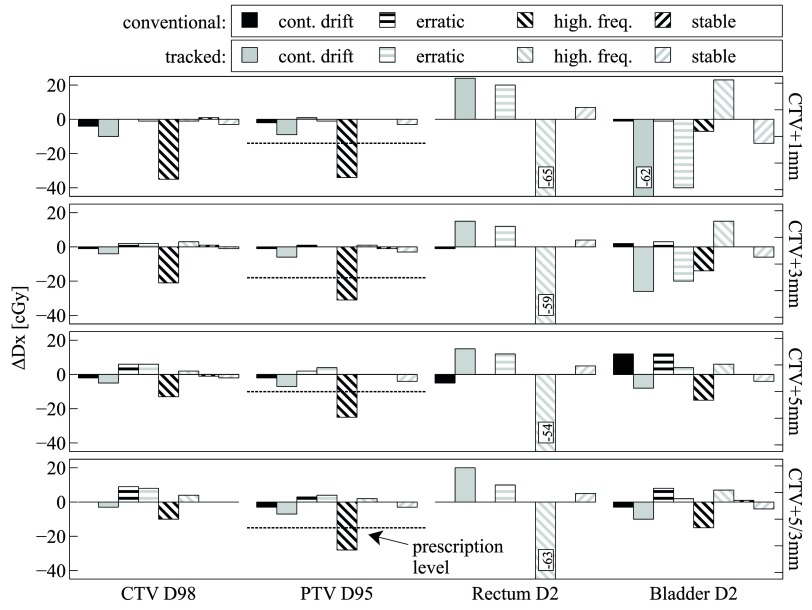
Patient 1: all results are relative to the respective Dx from the *static* deliveries. Results for each trajectory always come in pairs of *conventional*/*tracked*. Note that some dose differences are very small and thus not visible and that some bars are cropped.

**Figure 3.**
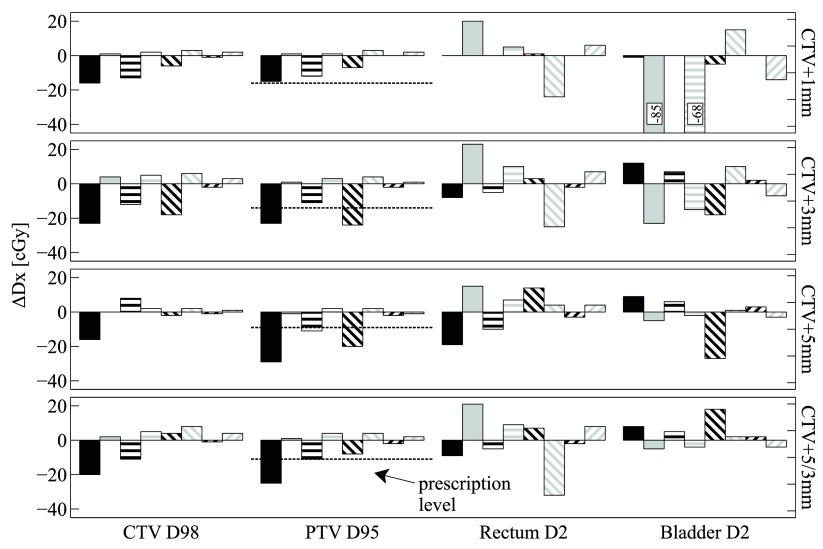
Patient 2: all results are relative to the respective Dx from the *static* deliveries (see legend and caption of figure 2).

**Figure 4.**
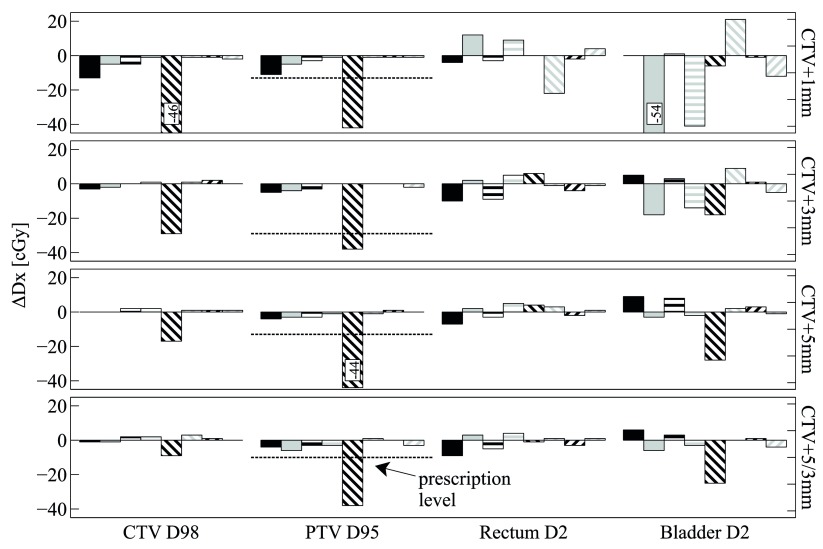
Patient 3: all results are relative to the respective Dx from the *static* deliveries (see legend and caption of figure 2).

Figures 2–4 summarise the findings for all patients and all combinations of plan and motion parameters. Importantly, all dosimetric results Dx are shown relative to their respective static case, which was considered the RTOG-compliant gold standard delivery. Results are always shown for a single fraction only, as intrafractional motion can vary strongly between fractions. The D95 prescription level (725 cGy for a single fraction) is indicated for the PTV.

For patient 1 (figure 2), the *high frequency* trajectory has a major impact on the target coverage for the *conventional* delivery. Substantial cold spots appear in the CTV and the PTV. The latter structure violates its RTOG prescription threshold for all margins. Tracking is able to recover the target dose in all these cases. In terms of the OAR D2 doses, tracking introduces a tradeoff between the rectum and the bladder. Predominant motion towards the rectum (*continuous drift*) increases rectum dose and decreases bladder dose. For the *high frequency* trajectory, which moves predominantly towards the bladder, the trend is reversed. It it instructive to note that for the CTV+1 mm *tracked* deliveries, rectum and bladder dose increases lead to absolute dose values on par or below the respective values from the CTV+5/3 mm *static* delivery.

For patient 2 (figure 3), cold spots appear in the target in the *conventional* delivery for the *continuous drift*, the *erratic* and the *high frequency* trajectories. Interestingly, there is no clear trend for a reduction in CTV cold spots with margin. Again, tracking successfully removes these cold spots for all cases. For the OAR D2 in *tracked* deliveries, the tradeoff between rectum and bladder depending on the motion trajectory, as previously seen in patient 1, is repeated. When the CTV+1 mm *tracked* deliveries are compared to the CTV+5/3 mm *static* delivery, rectum D2 sometimes exceeds the *static* delivery whereas bladder D2 is lower in all cases.

Lastly, for patient 3 (figure 4), it is again the *high frequency* trajectory that results in large target cold spots for the *conventional delivery*. It is noticeable that the CTV cold spots reduce with margin increase, whereas the PTV cold spots do not. For the CTV+5 mm plan, the PTV D95 falls short of the RTOG prescription by 31 cGy. As seen for some plans of patient 2, the *high frequency* trajectory leads to a substantial reduction in bladder D2 for the *conventional* delivery. This is due to the fact that patient 3 (just like patient 2) has a relatively short target-to-bladder distance and that the *high frequency* trajectory is moving predominantly towards the bladder. Dose which is usually deposited into the bladder is thus received by the target instead. Similar to patient 1, rectum and bladder dose increases for the CTV+1 mm *tracked* deliveries lead to absolute dose values on par or below the respective values from the CTV+5/3 mm *static* delivery.

### 3.2. Computing performance

The online dose reconstruction was updated for each MLC aperture reported by the linac (25 Hz) and took $8.8\pm 1.0$ ms (min: 7.5 ms, max: 12.9 ms) per aperture at the $5\times 5$ mm^2^ beamlet resolution. Beamlet data of up to 1 GB per segment was handled at a memory throughput of $75.6\pm 8.5$ GB sec^−1^ (min: 51.2 GB sec^−1^, max: 87.6 GB sec^−1^) on a single workstation computer. Memory throughput was observed to be roughly constant for finer beamlet resolutions, resulting in dose reconstruction times increasing linearly with total beamlet data. The typical total number of dose calculations was of the order of 8000 for a 5.3 min delivery.

### 3.3. Impact of beamlet resolution

**Figure 5.**
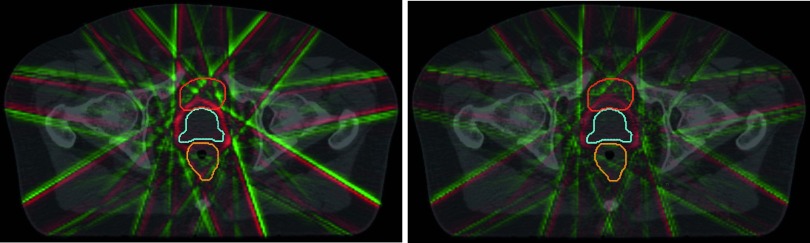
Dose calculated from $5\times 5$ mm^2^ beamlets minus dose calculated from $5\times 1.25$ mm^2^ beamlets for patient 2 and CTV+1 mm for a *tracked* delivery with *continuous drift* motion (left) and for a static delivery (right). The colour scale is set between  +25 cGy (green) and  −25 cGy (red).

The effect of the different beamlet resolutions is shown in figure 5. All dose differences were calculated relative to the $5\times 1.25$ mm^2^ beamlet resolution which was assumed to be the gold standard. The dose difference is most pronounced at the edge of the segments and therefore also in an annulus around the PTV. It is noteworthy that for the $5\times 1.25$ mm^2^ beamlet resolution, the dose distribution is ‘tighter’, meaning that dose is shifted from just outside of the segment (in beam’s-eye-view) to just inside the segment. This is due to the fact that in the case of a partial overlap of the MLC leaf with a beamlet, dose is distributed equally across this beamlet which reduces the spatial resolution of the dose distribution.

The dose differences are characterised in terms of the 5% and 95% percentile for a sub-sample of all settings in table 2. Although the maximum dose deviation in a single organ was found to be 25 cGy (in bladder) and 39 cGy in the entire body, this represents an outlier and is not a good proxy for the similarity of the two dose distributions. The standard deviation error was also deemed insufficient in describing the dose difference as it did not follow a normal distribution. For all cases investigated, the dose difference appears quite small, only of the order of a few cGy.

**Table 2.** Dose difference due to beamlet resolution in terms of 5% and 95% percentile for patient 2 and the CTV+1 mm plan.

Percentile	Static		Tracked (cont. drift)	
	5% [cGy]	95% [cGy]	5% [cGy]	95% [cGy]
Dose difference: $5\times 5$ mm^2^ D_*ij*_ –$5\times 1.25$ mm^2^ D_*ij*_				
CTV	−3.4	1.3	−4.5	1.2
Rectum	−4.9	5.2	−7.5	6.6
Bladder	−6.0	5.0	−6.5	6.0
Dose difference: $5\times 2.5$ mm^2^ D_*ij*_ –$5\times 1.25$ mm^2^ D_*ij*_				
CTV	−1.7	0.4	−1.7	0.1
Rectum	−2.1	1.9	−3.0	2.6
Bladder	−2.1	1.8	−2.4	2.1

When analysing the DVH curves for the different VOIs, the beamlet resolution caused no discernible difference. This was to be expected from the dose difference distributions and the small magnitude of dose difference.

### 3.4. Comparison with rigid shift method

**Figure 6.**
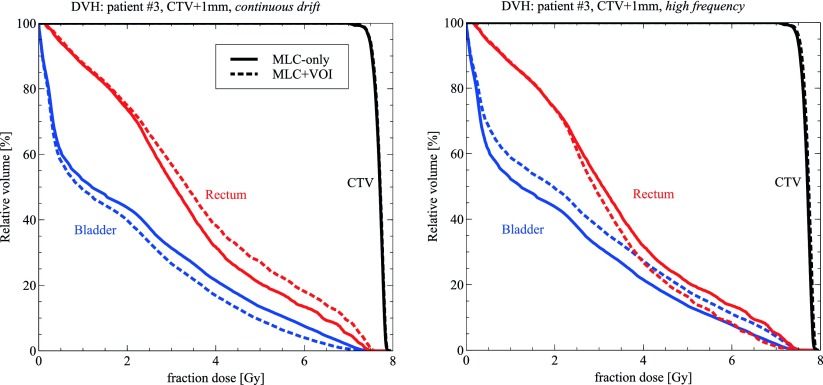
Impact of reconstruction technique on dose for two sample cases. *MLC+VOI* denotes the technique established in this study. *MLC-only* is an alternative approach leaving the target VOI static.

When comparing the *MLC+VOI* translation method used throughout this study with the simpler *MLC-only* translation method (see section 2.3) for a small number of sample cases (figure 6), it becomes obvious that both methods produce very similar dose distributions across the CTV. For the OARs, however, the difference is pronounced and the *MLC-only* translation method either over or underestimates the OAR dose depending on the most prominent direction of motion. For example, for the *continuous drift* trajectory which steadily drifts posteriorly, the rectum D10 dose increases by 50 cGy when comparing the *MLC-only* with the *MLC+VOI* reconstruction.

### 3.5. Dosimetric impact of interfractional motion

**Figure 7.**
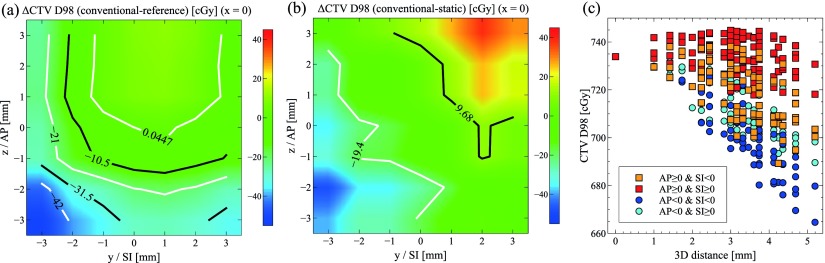
Effect of interfractional and intrafractional motion on CTV D98 for a *conventional* delivery (patient 3, CTV+1 mm, *continuous drift*) in the sagittal plane ((a)–(b)) and in 3D (c).

Figure 7 demonstrates the effect of residual beam-target misalignment caused by interfractional motion for the *conventional* delivery. When comparing CTV D98 as a function of interfractional offset with the zero-offset reference case, i.e. the idealised *conventional* delivery with *x*  =  *y*  =  *z*  =  0, it becomes obvious that intrafractional motion can compensate for interfractional offsets (figure 7(a)). The top right quadrant, indicating superior and anterior interfractional offsets, is largely free of dose deviations as the prostate drifts back to the centre during the delivery for the *continuous drift* trajectory. Additionally, CTV D98 for the offset-shifted *conventional* deliveries subtracted from the respective offset-shifted *static* delivery is shown in figure 7(b). This allows to isolate the effect of intrafractional motion. Interestingly, a large swath of interfractional position offsets is relatively robust against intrafractional shifts. This confirms the relatively small effect of *continuous drift* motion on the CTV during the *conventional* delivery as seen in figure 4. Figure 7(c) confirms for all possible combinations of 3D shifts that a combined anterior and superior interfractional shift yields the highest CTV D98 dose, whereas a combined posterior and inferior shift yields the lowest doses on average.

### 3.6. Impact of prostate rotations

**Figure 8.**
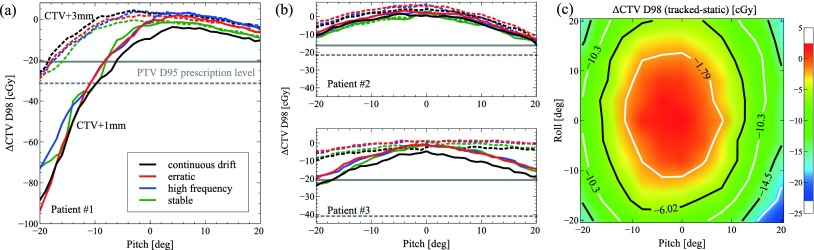
*Tracked* CTV D98 minus respective (unrotated) *static* CTV D98: ((a)–(b)) as a function of the pitch rotation applied to the prostate for all three patients, and (c) as a function of pitch and roll rotations for patient 2 (CTV+1 mm, *high frequency*). Solid (dashed) curves correspond to the CTV+1 mm (CTV+3 mm) plan.

Figures 8(a) and (b) highlights the impact of prostate rotations about the LR axis (pitch) on the CTV D98 dose for *tracked* deliveries. It is important to keep in mind that during the *tracked* delivery, only the prostate translations are explicitly compensated for, whereas rotations are not taken into account. Overall, the effect of the rotation appears to outweigh the effect of the intrafractional motion, especially for larger angles of rotation. Plans using the smaller 1 mm margin appear less robust against rotations than plans with 3 mm margin. For patient 1, negative pitch rotations result in a dramatic reduction of CTV D98 compared to the static reference. At rotation angles smaller than $-{{10}^{{}^\circ}}$, CTV D98 even drops below the PTV D95 prescription of 725 cGy, indicating areas of cold spots for the CTV+1 mm plan. For the same patient, the additional effect of simultaneous rotations about the SI axis (roll) appears considerably smaller than the effect of pitch-only rotations. For patients 2 (figure 8(c)) and 3, roll and pitch rotations have a similarly detrimental effect on CTV coverage.

## 4. Discussion

We successfully implemented real-time dose reconstruction for experimental treatment deliveries on a research linac. In terms of the algorithmic performance, our dose reconstruction achieves an average memory throughput of 75.6 GB sec^−1^, which compares very favourably with the 74.5 GB sec^−1^ measured using the STREAM benchmark^1^1http://www.cs.virginia.edu/stream/ (version 5.10) compiled using gcc 4.8. commonly used for bandwidth-limited applications. This indicates that run-times reported here are scalable and that further speed-ups are to be expected with improved hardware. Most computations were performed using a default beamlet resolution of $5\times 5$ mm^2^. Theoretically a finer sampling of the beamlets in the direction parallel to the MLC leaves is beneficial, although the finite voxel resolution of the dose grid will counteract some of that benefit. To that effect, we have also tested $5\times 1.25$ mm^2^ and $5\times 2.5$ mm^2^ beamlet resolutions in offline reconstruction mode for a *static* and a *tracked* delivery. For both deliveries and both beamlet resolutions, the DVHs of CTV and OARs remain unaffected by some small local dose deviations of a few cGy. A finer sampling in the direction orthogonal to the MLC leaves could increase the accuracy for beamlets partially covered by the Y collimators, but was not tested as it is not expected to be beneficial for the majority of MLC leaves as beamlets and leaves both have a 5 mm width (at the isocenter) and are perfectly aligned.

### 4.1. Dosimetric results

For three patients and combinations of four plans and four realistic target motion trajectories, our reconstructed dose distributions highlight the fact that target dose in a single fraction measured in either CTV or PTV can be safely recovered using dynamic MLC tracking. This is especially relevant for occasional ‘outlier’ motion events such as the ones seen in the *high frequency* trajectory. It is important to note that dose recovery in the target, i.e. the removal of cold spots, is also observed for the smallest investigated margin of 1 mm. We selected this lower margin limit to account for residual tracking errors caused by system latencies and target detection uncertainties (Fast *et al* 2014). Whether such small margins are really feasible depends, amongst other things, on the level of confidence in the accuracy of modern delivery systems. The limited conformity of photon radiotherapy planning due to the shallow dose fall-off might in this case be desirable, as it takes care of small motion events and some of the microscopic spread. Although high conformity is an objective during plan optimisation, the V95% isodose line still extends well beyond the PTV in many regions. In terms of the OAR D2 dose, MLC tracking induces both reductions and increases depending on the motion model. This could become a concern if clinical dose constrains are violated, something which potentially requires a plan adaptation. This study also shows that in the case of large prostate rotations, the use of reduced margins without rotating the collimator and/or adapting the beam aperture, is potentially unsafe and needs further investigation. Deutschmann *et al* (2012) have reported mean interfractional rotations about LR of $5.3\pm {{4.9}^{{}^\circ}}$ with a maximum rotation of 30.7°, and mean intrafractional rotations of $2.5\pm {{2.3}^{{}^\circ}}$ (maximum: 26.9°), highlighting the significance of these rotations. Their study followed in the footsteps of earlier work at the NKI-AvL (Hoogeman *et al* 2005, Rijkhorst *et al* 2007), which strongly suggested that rotations can be dosimetrically significant, sometimes more so than translations, and need to be adapted to.

Special emphasis was put on interfractional motion for the *conventional* delivery. It was shown that for the tight 1 mm CTV-to-PTV margin, the residual beam-to-target misalignment can have an impact on the dose distribution and assuming perfect target setups (as was done for most of this study) might make the *conventional* delivery look slightly better than it really is. For the tracked delivery it is assumed that small interfractional offsets can be perfectly compensated for by MLC tracking as the motion monitoring device does not differentiate between intrafractional and interfractional motion.

Our results agree well with results for the first clinical prostate tracking trial presented by Colvill *et al* (2015). Using the retrospective dose reconstruction method developed by Poulsen *et al* (2012), the authors show for 15 patients and 475 suitable treatment fractions that MLC tracking improves the consistency between planned and delivered dose and recovers dose during ‘outlier’ fractions with significant motion events well. In contrast to our study, the authors used a dual-arc volumetric delivery and a mix of conventional fractionation and hypofractionation. For our study, we developed a second dose reconstruction similar to the rigid shift method developed by Poulsen *et al* (2012) (see section 2.3), the main difference being that we did not group motion into discrete intervals. We noticed almost identical results for the target and different results for the OARs which can be explained by the different assumptions about OAR motion (rigidly shifted with target versus static).

Other offline reconstruction approaches for MLC tracking focus on gamma pass/failure rate as a quality indicator (Ravkilde *et al* 2013, 2014). Ravkilde *et al* (2014) have presented a framework in which dose is calculated using a custom-made ‘motion-including’ pencil beam convolution algorithm and they verified it against a temporally and spatially resolved dose measurement from a biplanar dosimeter. Good agreement was shown when comparing the computed dose with the measured dose. Despite several simplifying assumptions in the dose calculation, the mean dose calculation time was reported as 259 ms with a temporal resolution of 500 ms. This is significantly slower and less frequent compared to our study. It is often implied that the *static* dose delivery constitutes the *gold standard* delivery which needs to be matched or improved upon in any adaptive delivery. While this is intuitively correct, there are many situations in which this assumption cannot be fulfilled, e.g. when the prostate-to-OAR distance decreases, resulting in a change of anatomy. In this study we chose to compare VOI-specific dose characteristics with the *static* dose delivery instead of using global measures like the gamma pass/fail rate. This is to reflect the reality of treatment planning which is based on a set of VOI-specific dose constraints and objectives.

MLC tracking, by design, is only intended to remove the motion-related portion of the margin. This includes not only intrafractional motion which contributes relatively little to the overall margin according to van Herk’s margin recipe (van Herk 2004) based on population statistics, but also the proportion of the margin needed for interfractional motion, or to be more accurate, residual interfractional motion after employing modern image guidance and couch shifts. It cannot reduce other margins such as those related to delineation uncertainty. Generally, small prostate margins are met with scepticism since (Engels *et al* 2009) highlighted the dangers of shrinking margins. It should be noted, however, that their study did not use MLC tracking to conform the target dose. Another point of criticism might be the microscopic tumour spread of tumour tissue into adjacent normal tissue. Sohayda *et al* (2000) observed extracapsular extension (ECE), seen even in the earliest stages of localised prostate cancer, in 35% of all investigated prostatectomy specimens. While the mean ECE was 1.1 mm, a maximum extension of 1 cm was also observed with the majority of cases orientated in the posterolateral direction.

### 4.2. Limitations and outlook

In this study we relied on certain DVH points such as CTV D98 to characterise entire dose distributions. This simplification was necessary in order to reduce the vast amount of data contained in the DVHs, but the exact choice of a DVH point is somewhat arbitrary. Throughout this study we limited ourselves to the analysis of single fractions, to exclude any averaging effects over multiple fractions with potentially different intrafractional motion in each fraction.

Further limitations of this study are the use of the singular value decomposed pencil beam algorithm and more broadly speaking the use of pre-calculated dose influence data. While the pencil beam algorithm is acceptable for prostate, other sites such as lung or liver would profit from algorithms better at handling tissue inhomogeneities, such as collapsed-cone or Monte Carlo. Due to the modular design of our dose reconstruction software, the choice of physical dose engine is flexible and one can be exchanged for the other without affecting the calculation time. This is especially relevant for applications on MR-guided treatment machines where Monte Carlo dose calculation under consideration of the magnetic field is essential. The reliance on pre-calculated beamlets might be problematic for very small MLC apertures due to the output factor effect, although we tried to avoid such segments in this study. MLC leakage and the tongue-and-groove effect were also not accounted for in the beamlets, as leakage was previously measured to be below 0.2% and the Agility MLC has defocused leaves without a physical tongue-and-groove (Bedford *et al* 2013). Additionally, more complex motion might see the original planning CT completely invalidated. In those cases, pre-calculation of dose is impossible. New 3D planning images would need to be acquired in treatment position either directly or indirectly through motion models. Very fast Monte Carlo simulations such as the one presented by Ziegenhein *et al* (2015) could become just about fast enough for a real-time application in the near future. One way to ease the computational burden would be to reduce the number of dose calculations according to the observed motion. In this study, we selected a reconstruction frequency of 25 Hz to capture each MLC aperture reported by the linac. It is conceivable that this number can be vastly reduced in times when the prostate motion is small without compromising the dosimetric accuracy.

Future work should also focus on developing more realistic motion models and assessing the impact on the actually delivered dose distribution. This is especially important for the OARs, which were considered static for the purpose of this study. In future, MR or in some circumstances ultrasound images are expected to prove useful for providing real-time position updates not only for the target but also for the OARs. In our software, motion models for individual OARs could easily be added without significantly increasing computing times. Experiments using deformable phantoms or digital phantoms would then provide a useful way of validating the motion model and measuring the actually delivered dose.

This study clearly showns that MLC tracking can change the dose received by normal tissues compared to the original treatment plan. This inadvertent change of the fractionation might have unintended consequences in terms of the biological effects of normal tissue repair and warrants further investigation. Based on the software infrastructure laid out in this study, it should also be possible to investigate novel treatment schemes such as the ‘isotoxic’ approach in which the target dose is boosted until the OAR dose of a small margin plan is equivalent to the OAR dose of a large margin plan.

## 5. Conclusion

In this study, we have demonstrated that real-time and online dose reconstruction is feasible when harnessing modern workstation computers without compromising on spatial resolution or temporal accuracy. We believe that online dose reconstruction will become an important tool for creating confidence in tracked treatments. This is also an important milestone towards the implementation of online re-planning for adaptive radiation therapy and dose-guided tracked MLC deliveries. Limitations of our study are the use of pre-calculated dose influence data and the simplistic anatomical model. Keeping these limitations and the small number of patients in mind, initial evidence has been given that MLC tracking of prostate translations with smaller margins than currently recommended could allow for sufficient CTV coverage. Changes of OAR dose occur during MLC tracking and are non-negligible. Our results also indicate that prostate rotations can have a significant dosimetric effect on the target and should be considered in future work.
